# 

**Published:** 1849-01

**Authors:** 


### INDEX.

Abnormal position of the viscera, case of, 512
Abscess, in the prostate gland, 200
" in the perineum, 198
" of the uterine appendages, by Dr. Bennet, 75
" of right corpus striatum, 556
Absorption of insoluble bodies, 43
Acetone, use of, in cholera, 457
Action of chloroform on the sensitive plant, 374
Action of the pancreatic fluid. By M. Ch. Bernard, 40
Albumen, presence of, in the urine. By Dr. Finger, 326
Alkalies, utility of, in the treatment of rheumatism. By J. I. Furnivall, M. D. 134
Ammonia, carbonate of, in squamous affections of the skin, 67
Amputation at the shoulder-joint, successful case of, 451
Amputation, successful, of the thigh, at the hip-joint, 138
Anæsthesia, clinical observations upon. By Dr. Beau, 51
Anæsthesia, or the employment of chloroform and ether in surgery, midwifery, &c. By J. Y. Simpson, M. D., etc., bib. notice of 317
Anæsthetics in midwifery, use of. By J. Y. Simpson, M. D., of Edinburgh, 205, 269
Analysis, chemical, of cod liver oil, 400
Anaphrodisiac nature of lupulin, 284
Anatomical injections, new material and process for making minute, by Paul. B. Goddard, M.D. 719
Anatomy and physiology, cyclopædia of, edited by Robert B. Todd, M. D., F.R.S., bib. notice of, 469
Anatomy, human, an illustrated system of, special, general, and microscopic. By Samuel George Morton, M. D., bib. notice of, 97
Anatomy, human, by Jones Quain, M. D., bib. notice of, 426
Annual report of the officers of the New Jersey state lunatic asylum at Trenton, December, 1848, bib. notice of, 260
Antidote to strychnia, 46
Antidotes to opium and other narcotic poisons, 61
Application of the subcutaneous section to the treatment of lipoma, 137
Appointments, 371, 692
Appointments, naval, 38
Arachnoid, ossification of 382
Army medical department, 266, 488, 548, 752
Arsenic, cases of poisoning by, 559
Arsenic, detection of, in minute quantity, by T. F. Geoghegan, M. D. 43
Arterial compression in inflammation of the extremities, by Dr. Francois Henroz, 389
Arterial movement of the brain, 127
Arthritis in connection with uterine diseases, by M. Malgaigne, 140
Asiatic cholera, successfully treated by chloroform, 459
Association, American medical, 124, 356
Association, Am. medical, standing committees of, for 1849–'50, 435
Asphyxia, singular case of, 594
Astragalus, compound fracture and dislocation of, 69
Auscultation and percussion, a manual of, by MM. Barth & Roger, translated with additions, by Francis G. Smith, M. D., Philadelphia, second edition, bib. notice of, 362

Baillarger on monomania, 511
Beau's clinical observations upon anæsthesia, 51
Bedstead, description of a, for the treatment of fractures on board ship, by W. F. Jackson, M. D. 329
Bell on the relation of cholera to malarial fever, 448
Bennet on inflammation and abscess of the uterine appendages, 75
Bernard on the action of the pancreatic fluid, 40
Betton's case of asphyxia, 594
Bibliographical notices, 25, 97, 162, 236, 299, 343, 405, 469, 541, 600, 662, 731
Births, monstrous, succession of, occurring in the same female, 23
Bladder, case of calculi in, 390
Blockley almshouse, cholera in, 490
Board of examiners, 622
Board of examiners, U. S. army, 267
Bougies and catheters, preservation of, 546
Brain, arterial and respiratory movements of, 127
Brain, epilepsy from pressure upon, 648
Burns' case of placenta prævia, 720

Calculi in the prostate, 201
Calculi in the bladder, case of, 390
Camphor in solution, new vehicle for holding, 136
Camphor and chloroform mixture, note on a, 66
Carbon, existence of free, in the human body, 383
Carbonate of ammonia in squamous affections of the skin, by M. Cazenave, 67
Card, 34
Cases, surgical, communicated by T. D. Mutter, M. D. 226
Cases, surgical, by J. Steiner, M. D. 13, 391
Catheters, gum elastic, method of preserving, in warm climates, 621
Catheters, preservation of, 546
Cauterization of cancerous degeneration of the os uteri, 139
Cerebral congestion in relation to hemorrhage and ramollissement of the brain, 52
Cerebro-spinal meningitis occurring during the prevalence of epidemic catarrhal fever, by R. L. Scruggs, M. D. 17
Chancre, gonorrhœa, &c., action of the proto-sulphate of iron in the treatment of, 136
Changes, professorial, 320
Changes which take place in the lungs after division of the pneumogastric nerves, by Dr. Schiff, 373
Charge of Judge Burnside, and speeches of defendants' counsel, in the case of Hinchman vs. Ritchie, et al, bib. notice of, 473
Chemical analysis, qualitative and quantitative, by Henry M. Noad, lecturer on chemistry at St. George's hospital, etc. etc., bib. notice of, 349
Chemical and pharmaceutic manipulations, by Campbell Morfit, bib. notice of, 105
Chemical changes of respiration, 374
Chemical nature of cod liver oil, 500
Chemical technology, or chemistry applied to the arts and manufacfactures, by Dr. F. Knapp, professor of the university of Geissen etc. etc., bib. notice of, 434
Chemistry, encyclopædia of, theoretical and practical, presenting a complete and extended view of the present state of chemical science, by Jas. C. Booth, member of the American philosophical society, &c. &c., 101
Chemistry of vegetable and animal physiology, by G. J. Mulder, professor of chemistry in the university of Utrecht, bib. notice of, 469
Chemistry, practical, an introduction to, including analysis, by John E. Bowman, demonstrator of chemistry in King's college, London, bib. notice of, 182
Children, the maternal management of, in health and disease, by Thomas Bull, M. D., member of the royal college of physicians, &c. &c., bib. notice of, 236
Chlorate of potash in the treatment of erysipelatous inflammations of the mouth and fauces; vulgo, black tongue, by R. L. Scruggs, of Germantown, Tenn. 227
Chloride of Zinc, recovery from poisoning with, and the suggestions of an antidote for this poison, 128
Chloroform in traumatic tetanus, 137
Chloroform, use of, in insanity, by Dr. McGavin, 56
Chloroform in natural labor, by R. P. Stevens, M. D., of Ceres, Penna., 572
Chloroform, action of, in the sensitive plant, 374

Chloroform and ether in puerperal insanity, 447
holera, 117
Cholera, absence of precursory diarrhœa in, 507
Cholera, Asiatic, successfully treated by chloroform, 459
Cholera, case of, at Cape May, 641
Cholera, cryptogamous origin of, 683
Cholera, diet in, 585
Cholera, directions relative to the prevention and treatment of, 48
Cholera, disappearance of, from Philada., 545
Cholera, epidemic, treatise on, by Ambrose Tardieu, M. D., &c., bib. notice of, 405
Cholera in Blockley almshouse, history of, 650
Cholera in Philadelphia, 438, 489
Cholera, its relation to malarial fever, 448
Cholera, progress of, 372
Cholera, progress of, in the United States, 35
Cholera, report on, in Paris, bib. notice of, 482
Cholera, saline treatment of, 683
Cholera, sedative treatment of, 525
Cholera, use of acetone in, 457
Cholera, use of iron as a prophylactic against, 68
Classes, medical, 1848–9, 372
Clift, Mr., F. R. S., obituary of, 487
Clinical midwifery, by Robert Lee, M. D., F. R. S., etc., bib. notice of, 108
Clinical notes from private practice, 575
Cod liver oil, chemical analysis of, 400
Cod liver oil, general results of the use of, in pulmonary consumption, 440
Cod liver oil, mode of operation of, 442
Cod liver oil, preparation and administration of, 445
Cod liver oil, three kinds of, comparatively considered with reference to their chemical and therapeutical properties, by L. J. de Jongh, M. D., bib. notice of, 746
Cod liver oil, use and administration of, in pulmonary consumption, by C. J. B. Williams, M. D., F R. S , &c. &c. 439
Collodion as a means of arresting hæmorrhage from leech-bites, 134
Collodion, observations on, in the treatment of diseases of the skin, 58
Colon, intussusception into the, 131
Color of the hair, 499
Compound fracture and dislocation of the astragalus, 69
Constitutional syphilis, 71
Convention, national, for revising the pharmacopœia of the U. S., 322
Cooper, Mr. Samuel, death of, 123
Corpus striatum, abscess of right, 556
Cours de physiologie fait à la faculté de médecine de Paris, par P. Bérard, professeur de physiologie à la faculté de médecine de Paris, bib. notice of, 469
Cure of cancers, 60
Cutaneous disease, localisation of, 132
Cryptogamous origin of cholera, 683
Cryptogamous origin of malarious and epidemic fevers, by J. K. Mitchell, A. M., M. D., bib. notice of, 238
Cyanosis, Rokitansky on, 218
Cyclopædia of anatomy and physiology, edited by Robert B. Todd, M. D., F. R. S., 469
Death of Mr. Samuel Cooper, 123
Death of prof. Barbour, of St. Louis, 486
Delegates, 189
Delivery impeded by an intra-pelvic tumor, case of, 455
Deontology, medical : or concerning the duties and rights of physicians in the present state of civilization, by Dr. Max Simon, bib. notice of, 732
Detection of arsenic in minute quantity, 43
Diabetes, cause of, 54
Dictionary of dental science, biography, bibliography, and medical terminology, by Chapin A. Harris, M.D., D.D.S., &c. &c., bib. notice of, 317
Diet in cholera, 585
Digitaline, 68
Diphtheritic inflammation of the pharynx and tonsils, 335
Directions relative to the prevention and treatment of cholera, 48
Disease, ocular, case of, 41
Diseases of the prostate gland, 199
Diseases of women, 73
Dislocation of the inferior surface of fifth cervical vertebra, reduction of, 72
Dislocation of the femur on the dorsum ilii, in a child aged three years and a half, 70
Displacements of the uterus, 73
Dissertation on the practice of medicine, by Tomlinson Fort, M. D., bib. notice of, 541

Dowler on vital dynamics, 612
Dysentery, epidemic, by Samuel Jackson, M. D., (late of Northumberland,) 701

Ear, use of glycerine in diseases of, 560
Early pregnancy, 447
Editorial, 34, 117, 183, 266, 318, 356, 435, 485, 545, 683, 751
Editorial changes, 488
Electricity, notes on the medical application of, by Wm F. Channing, M. D., bib. notice of, 316
Elements of materia medica and therapeutics, by Jonathan Pereira M. D., &c., bib. notice of, 162
Encyclopædia of chemistry, by Jas. C. Booth, member of the American philosophical society, &c., bib. notice of, 101
Enlargement of the prostate in old age, 202
Epidemic cholera in Philadelphia, 615
Epidemic cholera in Blockley almshouse, history of, 650
Epidemic dysentery, by Samuel Jackson, M. D., (late of Northumberland,) 701
Epilepsy from pressure upon the brain, 648
Erysipelas attacking the mucous membrane of the lungs, 395
Essays on infant therapeutics, by Jno. B. Beck, M. D., bib. notice of, 25
Ether and chloroform, employment of, in surgery, midwifery, &c., by J. Y. Simpson, M. D., bib. notice of, 317
Ether, use of, in puerperal insanity, 447
Etherization, case of tetanus cured by, 388
Eversion of the lower eyelid, case of, 21
Evidences of poisoning by Prussic acid, 47
Exhaustion, death from, 390
Existence of free carbon in the human body, 383

Females, a treatise on the diseases and special hygiene of, by Colombat de l'Isere, translated by Chas. D. Meigs, M. D., &c., bib. notice of, 682
Femur, dislocation of, on the dorsum ilii, in a child aged three years and a half, 70
Fevers, modus operandi of quinine in, 538, 592
Finley on cholera, 398, 524
Fistula, vesico-vaginal, case of, relieved by the ordinary hare-lip suture, 155
Fistulous openings in the perineum, 198
Fort, T., M. D., practice of medicine, bib. notice of, 541
Fracture of the os femoris, compound, 399
Fractures of the lower extremities, bedstead for the treatment of, on board ship, 329
Fœtus, intra-uterine peritonitis in, 57

General paralysis, 229
Glycerine, use of, in diseases of the ear, 560
Goddard on a new material and process for making minute anatomical injections, 719
Gonorrhœa, action of the proto-sulphate of iron in the treatment of, 136
Gout, 194

Hæmatocele, remarks on a case of, 520
Hair, color of, 499
Hall on paroxysmal paralysis, 549
Handwörterbuch der physiologie mit kücksicht auf physiologische pathologie, u. s. w., herausgegeben, von Dr. Rudolph Wagner, prof. in Gottingen, 469
Heart and great arteries of a child, extraordinary malformation of, at birth, 192
Heart-clot, by Charles D. Meigs, M. D. 141
Heart, mechanism by which the sounds of, are produced, 323
Heart, needle found in, after death, 93
Hemorrhage and ramollisement of the brain, cerebral congestion in relation to, 52
Hemorrhage from leech-bites, collodion as a means of arresting, 134
Hernia, bad effects arising from taxis in, 387
Hernia of the uterus, 73
Hernia, strangulated, use of chloroform in the reduction of, 386
Hervieux on digitaline, 68
Hiccup, intention of, 42
Hidden seizures, by Dr. Marshall Hall, 697
Hooker's physician and patient; or a practical view of the mutual duties, relations and interests of the medical profession and the community, bib. notice of, 732
Hospital, Philadelphia, Blockley, 752

Hospital, Pennsylvania, 95, 159, 231, 294, 339, 401, 547, 595
Hospital, St. Joseph's, 546
Hufeland on the relations of the physician to the sick, to the public and to his colleagues, bib. notice of, 732
Human anatomy, by Jones Quain, M. D., bib. notice of 426
Human anatomy, illustrated system of, special, general, and microscopic, by S. G. Morton, M. D., bib. notice of, 97
Human parturition, a theoretical and practical treatise on, by H. Miller, M. D., &c., bib. notice of, 434
Humor, vitreous, structure of, 493
Hydatids, uterine, 204
Hydrocephalus, necroscopic examination of a case of, with hypertrophy of the walls of the cranium, 286
Hysteria, remarkable case of, 331

Incarcerated hernia, reduction of, 139
Infant therapeutics, essays on, by John B. Beck, M. D., bib. notice of, 25
Infantile menstruation, 494
Inflammation of the extremities, arterial compression in, 389
Injection of stimulants into the veins 644
Inoculation, manslaughter by, 38
Insane, report of the Pennsylvania hospital for the, for the year 1848, by Thos. S. Kirkbride, M. D., bib. notice of, 260
Insanity, puerperal, 327
Insanity, use of chloroform in, 56
Insoluble bodies, absorption of, 43
Intention of hiccup, 42
Intestinal mucous membrane, 125
Intestine, union of a wound in, returned without suture, 153
Introductory lecture to the course of clinical instruction in surgery at the Pennsylvania hospital, by Geo. W. Norris, M. D., bib. notice of, 113
Introductory lecture to the class of midwifery and the diseases of women and children, in Jefferson medical college, by Charles D. Meigs, M. D., bib. notice of, 115
Introductory lecture to the course of chemistry, delivered in Jefferson medical college, by F. Bache, M. D., bib. notice of, 116
Introductory lecture, before the anatomical class of the university of Pennsylvania, by Wm. E. Horner, M. D., bib. notice of, 111
Introductory lecture to a course on surgery, to the medical class of Geneva college, by Prof. Bryan, bib. notice of, 264
Introduction to practical chemistry, including analysis, by John E. Bowman, demonstrator of chemistry in King's college, London, bib. notice of, 182
Introductory lecture on the coinciding tendencies of medicines, by Jas. P. Kirtland, M. D., bib. notice of, 264
Introductory lecture, delivered to the class of institutes of medicine in Jefferson medical college, Oct. 19, 1848, by Robley Dunglison, M. D., bib. notice of, 29
Intussusception into the colon, 131
Iron, use of, as a prophylactic against cholera, 68

Jackson, Samuel, M. D., (late of Northumberland,) on epidemic dysentery, 701
Jones on the intestinal mucous membrane, 125
Journal, Transylvania medical, 692
Journal, southern medical and surgical, 124
Journal, St. Louis medical and surgical, 437
Journal, western, of medicine and surgery, 38

Knee-joint, wound of, followed by phlebitis, 292

Labor, chloroform in natural, 572
Lecture, introductory, by Wm. E. Horner, M. D., bib. notice of, 111
Lecture, introductory, by Geo. W. Norris, M. D., bib. notice of, 113
Lecture, introductory, delivered to the class of institutes of medicine in Jefferson medical college, by Robley Dunglison, M. D., bib. notice of, 29
Lecture, introductory, by Chas. D. Meigs, M. D., bib. notice of, 115
Lectures on the processes of repair and reproduction after injuries, by Jas. Paget, F. R. C. S., &c., 693
Lectures on diseases of infancy and childhood, by Chas. West, M. D., &c., bib. notice of, 675
Lectures on venereal and other diseases arising from sexual intercourse, by M. Ricord, bib. notice of, 238
Lectures on surgery, by Bransby B. Cooper, F. R. S., 198
Lecture, introductory, by Jno. Wiltbank, M. D., bib. notice of, 264
Lecture, introductory, by F. Bache, M. D., bib. notice of, 116
Lecture, introductory, by Prof. Bryan, bib. notice of, 264
Lectures on obstetrics and diseases of women and children, by Gunning S. Bedford, M. D., bib. notice of, 264
Lectures on the diseases of infancy and childhood, by Chas. West, M. D., bib. notice of, 675
Lectures on the processes of repair and reproduction after injuries, by Jas. Paget, 693
Lehrbuch der physiologie des menschen für aerzte und studirende, von Dr. August Friedrich Gunther, bib. notice of, 469
Lehrbuch der physiologie für studirende und aerzte, von D. Arnold Adolph Berthold, bib. notice of, 469
Lexicon, medical, of modern terminology; being a complete vocabulary of definitions, &c. &c., by D. Meredith Reese, M. D., bib. notice of, 31
Lime, alleged production of the phosphate of, in the egg during incubation, 376
Lithontripsy, Randolph's cases of, 288
Lithontripsy, two cases of, by the late Geo. McClellan, M. D. 513
Lipoma, application of the subcutaneous section to the treatment of, 137
Localisation of cutaneous disease, 132
Lungs, changes which take place in, after division of the pneumogastric nerves, 373
Lungs, erysipelas attacking the mucous membrane of, 395
Lupulin as an anaphrodisiac, remarks on, 284

Malarial fever, relation of cholera to, 448
Malarious and epidemic fevers, cryptogamous origin of, by J. K. Mitchell, A. M., M. D., bib. notice of, 238
Malformation of the heart and great arteries of a child at birth, 192
Manual of auscultation and percussion, by MM. Barth & Roger, translated by F. G. Smith, M.D., bib. notice of, 362
Manual of physiology, by W. Senhonsec Kirkes, M. D., bib. notice of, 170
Manslaughter by vaccination, 38
Maternal management of children, in health and disease, by Thos. Bull, M. D., bib. notice of, 236
Materia medica and therapeutics, elements of, by Jonathan Pereira, M. D., F. R. S., &c., bib. notice of, 162
McClellan on lithontripsy, 513
Medical association, 356
Medical association, American, 124
Medical application of electricity, notes on, by Wm. F. Channing, M. D., bib. notice of, 316
Medical department, U. S. army, 266, 488
Medical matters and medical men in London and Paris, notes of, by D. W. Yandell, M. D., bib. notice of, 109
Medical notes, 159
Medical society of the State of Pennsylvania, 268, 320
Medullary sarcoma in an infant, 138
Meeting of the Philada. county medical society, 124
Meigs on the heart-clot, 141
Meningitis, cerebro-spinal, occurring during the prevalence of epidemic catarrhal fever, 17
Menstruation, infantile, 494
Metastatic abscesses, 582
Midwifery, use of anæsthetics in, by J. Y. Simpson, M. D., 205, 269
Midwifery, clinical, by Robert Lee, M. D., F. R. S., &c., bib. notice of, 108
Midwifery, practical compendium of, by R. Gooch, M. D., bib. notice of, 353
Monomania, 511
Monstrous births, case of a succession of, occurring in the same female, 23
Morton, S. G., M. D., Anatomy, bib. notice of, 97
Movements of the brain, 127
Mucous membrane of the uterus after parturition, 75
Multiplication of vegetable cells by division, 39

Narcotic poisons and their antidotes, 61

National medical association, 267
Naval appointments, 38
Neck as a medical region, Hall on, 549, 622, 697
Necroscopic examination of a case of hydrocephalus, with hypertrophy of the walls of the cranium, 286
Needle found in the heart after death, 93
Negro woman, case of, who gave birth to twins of different color, 523
Note on a camphor and chloroform mixture, 66
Notes, medical, 157
Notes on medical matters and medical men in London and Paris, by D. W. Yandell, M. D., bib. notice of, 109

Obituary of Dr. Massenburg, 751
Obituary of Mr. Clift, F. R. S. 487
Obliteration of the right iliac vein, 74
Obstetrics, lectures on, by G. S. Bedford, M. D., bib. notice of, 264
Obstetrics, plea for, by John Wiltbank, M. D., bib. notice of, 264
Obstetrics, principles and practice of, by W. T. Smith, M. D., London, bib. notice of, 600
Obstetrics, the science and the art, by Chas. D. Meigs, M. D., bib. notice of, 299
Ocular disease produced by an electrical discharge, 41
Oil, cod liver, administration of, 445
Oil, cod liver, chemical analysis of, 400
Oil, cod liver, operation of, 442
Oil, cod liver, preparation of, 445
Oil, cod liver, three kinds of, comparatively considered, with reference to their chemical and therapeutical properties, by L. J. de Jongh, M. D., bib. notice of, 746
Opium and its antidote, 61
Opium, tolerance of, 68
Oration delivered before the South Carolina medical association, by P. C. Gaillard, M. D., bib. notice of, 431
Ossification of the arachnoid, 382
Os uteri, cauterization of, 139

Pancreatic fluid, action of, 40
Paralysis, case of general, 229
Paroxysmal paralysis, 549, 622
Parturition, a theoretical and practical treatise on, by H. Miller, M. D., bib. notice of, 434
Parturition, and principles and practice of obstetrics, by W. T. Smith, M. D., London, bib. notice of, 600
Pathology and therapeutics of cholera, 509
Pennsylvania hospital, 95, 159, 231, 294, 339, 401, 548
Pharmaceutical and chemical manipulations, by C. Morfit, &c., bib. notice of, 105
Peritonitis, intra-uterine, in the fœtus, 57
Perineum, fistulous openings in, 198
Perineum, abscesses in, 198
Perspiration, sanguineous, 381
Pharmacy, practical, by F. Mohr, Ph. D., &c. &c., bib. notice of, 343
Pharmacopœia of U. S., convention for revising, 322
Pharynx, diphtheritic inflammation of, 335
Philadelphia hospital, Blockley, resignation of principal in, 752
Philadelphia, the cholera in, 438, 489
Phlebitis, 74
Phlegmasia alba dolens, 74
Phosphate of lime and iron in the egg during incubation, 376
Phthisis, 194
Physician and patient; or a practical view of the mutual duties, relations and interests of the medical profession and the community, by Worthington Hooker, M. D., bib. notice of, 732
Physiology, a manual of, by W. S. Kirkes, M. D., &c., bib. notice of, 170
Pills, sulphur, 548
Placenta prævia, Dr. Burns' case of, 720
Plastic operation for the cure of eversion and distortion of the lower eyelid, 21
Poisoning by arsenic, 559
Poisoning with chloride of zinc, 128
Poisoning with pounded glass, unsuccessful attempt at, 46
Poisons, narcotic, 61
Potash, chlorate of, in the treatment of erysipelatous inflammations of the mouth and fauces, 227
Practice of medicine, a dissertation on, by T. Fort, M. D., bib. notice of, 541
Practice of surgery: embracing minor surgery, and the application of dressings, &c. &c., by J. Hastings, M. D., bib. notice of, 662
Practical treatise on the domestic management, and most important diseases of advanced life, by

Geo. E. Day, M. D., bib. notice of, 313
Practical compendium of midwifery by Robert Gooch, M. D., bib. notice of, 353
Practical anatomy, a text-book of, by Robert Harrison, M. D. &c., bib. notice of, 32
Pregnancy, early, 494
Professional services, 318
Progress of cholera in the United States, 35
Progress of cholera, 372
Prolapsus of the umbilical cord, 140
Proto-sulphate of iron in the treatment of chancre, gonorrhœa, &c. 136
Prostate gland, diseases of, 199
Prostate gland, abscess in, 200
Prostate gland, ulceration of, 201
Prostate gland, calculi in, 201
Prostate gland, enlargement of, in old age, 202
Prussic acid, evidences of poisoning by, 47
Puerperal insanity, 327
Pulmonary consumption, use of cod liver oil in, 439

Quantity of blood in the body, experiments upon, 459
Quinine, modus operandi of, in fevers, 538, 592

Ramollissement of the brain, 52
Randolph's cases of lithontripsy, 288
Reduction of incarcerated hernia, 139
Relations of the physician to the sick, to the public and to his colleagues, by the late Christopher Wm. Hufeland, M. D., bib. notice of, 732
Relation of cholera to malarial fever, 448
Repair and reproduction after injuries, 632, 693
Report of the Pennsylvania hospital for the insane, by Thos. S. Kirkbride, M. D., bib. notice of, 260
Report on the practical operation of the law relating to the importation of adulterated and spurious drugs, medicines, &c., by M. J. Bailey, M. D., &c., bib. notice of, 670
Resection of the scapula, 138
Respiration, chemical changes of, 374
Respiratory movement of the brain, 127
Resumé of experiments to determine the quantity of blood in the body, 459

Rheumatism, utility of alkalies in the treatment of, 134
Rokitansky on cyanosis, 218
Roux on metastatic abscess, 582

Sanguineous perspiration, 381
Sarcomatous tumor, containing hair and stearine, removed from the womb, 568
Sarcoma, medullary, in an infant, 138
Scapula, resection of, 139
Sensitive plant, action of chloroform upon, 374
Secretion, biliary, does calomel expel it? 196
Sedative treatment of cholera, 525
Simon's medical deontology: or concerning the duties and rights of physicians in the present state of civilization, bib. notice of, 732
Skin, collodion in the treatment of diseases of, 58
Small-pox appearing in a family, without a previous chance of contagion, 518
Speeches of defendants' counsel in the case of Hinchman vs. Richie, et al, bib. notice of, 473
Spleen, 40
Sounds of the heart, mechanism of, 323
Southern medical reports, 621
Southern medical and surgical journal, 124
St. Joseph's hospital, Philada., 510, 752
St. Louis medical and surgical journal, 437
Strangulated hernia, 386
Strictures of the urethra, new mode of dilating, 138
Strychnia, antidote to, 46
Successful amputation of the thigh at the hip-joint, 138
Suggestions on the treatment of cholera patients, addressed to the parochial boards jointly, by a committee of the royal college of physicians and the royal college of surgeons of Edinburgh, and Dr. Sutherland, the commissioner of the general board of health, 557
Sulphur pills, 548
Summary of the transactions of the college of physicians of Philadelphia, from September 6th, 1848, to January 2d, 1849, inclusive, 263
Summer medical schools of Philadelphia, 183
Surgery, lectures on, 198

Surgery, report of the committee on, 77
Surgical cases, J. M. Steiner, M. D. 13
Surgical cases communicated by T. D. Mütter, M. D. 226
Sydenham society's works, 188
Syphilis, constitutional, 71

Taxis in hernia, 387
Terminology, medical lexicon of, by D. M. Reese, M. D., bib. notice of, 31
Testicle, tumors of, 520
Tetanus caused by etherization, 388
Text-book of practical anatomy, by R. Harrison, M. D., bib. notice of, 32
Theory of the cause of diabetes, 54
Tigri on the spleen, 40
Tolerance of opium, 68
Trachelismus, 622
Traumatic tetanus, 137, 391
Treatment of cholera, 507, 524
Tumor, intra-pelvic, impeding delivery, 455

Ulceration of the prostate, 201
Umbilical cord, prolapsus, 140
Union of a wound in the intestine returned without suture, 153
Unsuccessful attempt at poisoning with pounded glass, 46
Upshur's clinical notes, 575, 637
Urethra, new mode of dilating strictures of, 138
Urine, presence of albumen in, 326
Uterus, displacements of, 73
Uterus, hernia of, 73
Uterus, mucous membrane of, after parturition, 75
Uterus, rupture of, 140
Uterine diseases, 140
Uterine hydatids, 204

Valves of the heart, 323
Vegetable cells, multiplication of, by division, 39
Vehicle for holding camphor in solution, 136
Veins, injection of stimulants into, 644
Venereal disease, lectures on, by M. Ricord, bib. notice of, 238
Verga on the spleen, 40
Vesico-vaginal fistula, case of, 155
Viscera, case of abnormal position of, 512
Vitreous humor, structure of, 493

Western journal of medicine and surgery, 38
Womb, sarcomatous tumor of, 568
Wound of knee-joint followed by phlebitis, 292
Wood naphtha in cholera, 457
Works of the Sydenham society, 188

Zinc, chloride of, cases of poisoning with, 128
To our readers, 751

